# Correlation between telomere shortening in maternal peripheral blood and fetal aneuploidy

**DOI:** 10.1186/s12884-023-06185-1

**Published:** 2024-01-02

**Authors:** Xiao-Xi Zhao, Le Le Bai

**Affiliations:** 1 Department of Gynecology and Obstetrics, Affiliate Hospital of Inner Mongolia Medical University, Hohhot, Inner Mongolia 010050 China; 2 First Clinical Medical College, Inner Mongolia Medical University, Hohhot, China

**Keywords:** Telomere length, Trisomy 21, Amniotic fluid, Lymphocytes

## Abstract

**Background:**

This study aimed to assess whether maternal telomere length is a more accurate predictor of trisomy 21 than maternal age while also exploring the factors influencing maternal and fetal telomere length.

**Methods:**

Forty mothers with fetuses carrying extra maternal copies of chromosome 21 were defined as trisomy 21 cases, and 18 mothers with normal karyotype fetuses were defined as controls. Telomere lengths of maternal blood lymphocytes and amniotic fluid cells were determined using real-time polymerase chain reaction. Fetal and maternal telomere lengths were compared between the two groups. Moreover, we analyzed the factors influencing maternal and fetal telomere length in the trisomy 21 pedigree. A logistic regression model was used to analyze the correlation between maternal telomere length and trisomy 21 risk. In addition, receiver operating characteristic (ROC) curve analysis was used to determine the accuracy of using maternal telomere length as an indicator of trisomy 21 risk.

**Results:**

The study revealed that both maternal and fetal telomere lengths were significantly shorter in trisomy 21 cases than in the controls. In the trisomy 21 group, the maternal age, occupation, and nationality showed no significant correlation with their telomere length; fetal telomere length exhibited a positive correlation with maternal telomere length. Furthermore, maternal telomere length shortening is associated with trisomy 21 (OR = 0.311; 95% CI, 0.109–0.885, *P* < 0.05). The results of ROC curve analysis indicated that a combined assessment of maternal age and maternal telomere length predicted fetal chromosome trisomy more effectively than a single assessment (area under the curve 0.808, 95% CI, 0.674–0.941, *P* < 0.001).

**Conclusion:**

Maternal age combined with maternal telomere length proved to be a superior predictor of trisomy risk. Additionally, maternal telomere length was found to influence fetal telomere length.

## Background

Telomeres are cap structures at chromosomal ends; their primary role is to maintain the stability and integrity of the chromosomal structure [1, 2]. Telomere length (TL) serves as a biological age marker [3], with its shortening indicating cellular senescence. Biological age offers a more accurate reflection of aging and risk of disease than actual age [4, 5]. TL can also function as a biological marker for assessing the risk of age-related diseases [6].

Approximately 90% of chromosome 21 nondisjunction errors occur in oocytes. This percentage primarily depends on the maternal age at the time of conception within the studied sample [7, 8]. The relationship between Down syndrome (DS) and telomere shortening has been reported previously. Albizua et al. [9] investigated the relationship between TL and nondisjunction of chromosome 21 in oocytes and their results showed that the TL of young mothers who gave birth to trisomy 21 fetuses was shorter than that of those who gave birth to diploid fetuses at the same age. Telomere shortening is a risk factor for chromosomal nondisjunction. Bhaumik et al. [10] compared changes in TL in women with DS and observed that telomeres naturally decrease in length with age. Individuals were classified by age (old, ˃35 years; and young, ˂ 35 years) and meiotic error (MI and MII). Old MII mothers displayed the shortest telomeres, while control mothers had the longest telomeres, and those of MI mothers lay in between. Ray et al. [11] investigated the relationship between telomere shortening caused by environmental factors and the occurrence of DS and noted that chewing tobacco caused telomere shortening and increased the risk of giving birth to children with DS. These findings suggest that maternal telomere shortening may elevate the risk of trisomy 21. Currently, indicators for pregnant women to undergo invasive prenatal diagnosis include maternal age at the time of conception, maternal serological screening results, fetal ultrasound results, and non-invasive prenatal testing (NIPT) results. Notably, the application of NIPT has effectively improved the detection rate of fetal chromosomal abnormalities [12]. However, NIPT is expensive and is not ideal as a first-line screening method—Liu et al. [13] reported that the positive predictive values of NIPT as a first-line screening method for trisomies 21, 18, and 13 were 78.46%, 62.96%, and 10%. For sex aneuploidies, the positive predictive value was 47.22%.

Hence, there remains a need to identify an affordable and effective screening method. In view of the current situation of prenatal screening and the relationship between telomeres and aneuploidy, we investigated the factors affecting maternal and fetal TL and the accuracy of using maternal TL as a predictor of a woman’s risk of having a child with DS.

## Methods

This study aimed to assess whether maternal TL is a more accurate predictor of trisomy 21 than maternal age while also exploring the factors influencing maternal and fetal TL.

### Participants

Patients who underwent amniocentesis at the Affiliated Hospital of Inner Mongolia Medical University in Hohhot, China, between January 2016 and December 2020 were selected as study participants. They were categorized into trisomy 21 and control groups based on the fetal chromosomal results.

The inclusion criteria for trisomy 21 were as follows: (1) confirmation of the standard trisomy 21 fetal chromosomal karyotypes, (2) enrollment of pregnant women who were healthy without pregnancy complications or a history of chronic diseases, and (3) identification of the extra chromosome 21 originating from the mother.

Inclusion criteria for the control group were as follows: (1) normal fetal chromosomal karyotype and (2) healthy pregnant women with no history of pregnancy complications or chronic diseases.

Exclusion criteria were as follows: (1) the presence of translocation or chimerism in the trisomy 21 karyotype and (2) healthy pregnant women with a history of unexplained adverse pregnancy outcomes.

Ethical approval was obtained from the Ethics Committee of the Affiliated Hospital of Inner Mongolia Medical University to collect general clinical data and amniotic fluid and blood samples (YKD-2016108). All selected participants provided informed consent.

### Basic clinical data collection and specimen collection

(1) Amniotic fluid and parental peripheral blood samples were collected from the 40 families with trisomy 21 at the same time. In the control group, only maternal blood and amniotic fluid samples were collected from 18 families with normal karyotypes at the same time. (2) Data regarding the women’s age, nationality (Han nationality or minorities), occupation, pregnancy week, and other information were further collected. (3) Amniotic fluid specimens were obtained: the supernatant was left for the amniotic fluid specimen after 1 week of culture and was subsequently cultured in another vial. Cultures were grown for 3–5 days, and the supernatants were aspirated when clones appeared. Subsequently, the supernatant was removed, an appropriate amount of normal saline was added, and the amniotic fluid cells were scraped from the bottle wall with a straw, centrifuged, and stored at -80 ℃. (4) Parental peripheral blood samples were collected with an Ethylene Diamine Tetraacetic Acid tube and stored at -80 ℃.

### TL measurement

After thawing the amniotic fluid cells and peripheral blood, DNA was extracted from amniotic fluid cells and peripheral blood lymphocytes. TL was determined using quantitative real-time polymerase chain reaction (PCR), as described by Cawthon [14]. Primers were synthesized by Shanghai Biotechnology Co., LTD.

The following primer sequences were used:

#### TEL 1

5’ – GGTTTTTGAGGGTGAGGGTGAGGGTGAGGGTGAGGGT – 3’.

#### TEL 2

5’ - TCCCGACTATCCCTATCCCTATCCCTATCCCTATCC-CTA – 3’.

#### 36B4u

5’ – CAGCAAGTGGGAAGGTGTAATCC – 3’.

#### 36B4d

5’ – CCCATTCTATCATCAACGGGTACAA – 3’.

Each sample was analyzed in triplicate and thermally cycled using a quantitative PCR, which was completed using a fluorescence quantitative PCR instrument. TL was expressed as the ratio of the duplicate copy number of the telomere sample to that of the control gene.

### Statistical methods

Statistical analyses were performed using SPSS 19.0 software (SPSS Inc., IL, United States). The age and gestational stage of the mothers in the trisomy 21 and control groups conformed to the normal distribution, and between-group differences were assessed using the t-test. The mother and fetal TL in the trisomy 21 and control groups did not exhibit a normal distribution; therefore, between-group differences were assessed using the Mann–Whitney U test. The proportions of occupations and nationalities of the mothers in the enumeration data were expressed as percentages. The chi-square test was used to compare the two groups. Binary logistic regression analysis was used to evaluate the association between maternal TL and the risk of trisomy 21. Using ROC curve analysis, the TL of maternal blood lymphocytes and maternal age were used as risk indicators to assess the accuracy of the trisomy 21 risk. Statistical significance was set at *P* < 0.05.

## Results

### Basic clinical data and TL of the participants

No significant difference was observed in gestational weeks, maternal occupation, or nationality between the two groups (*p* > 0.05). However, the average maternal age of the trisomy 21 group was significantly higher than that of the control group (*p* < 0.05). In both the study and control groups, maternal TL was consistent with fetal TL. In the control group, maternal TL was (1.42 ± 0.95) and fetal TL was (1.45 ± 0.93). Maternal TL was (0.85 ± 0.54) and fetal TL (0.84 ± 0.44) in the study group. The average TL in mothers and fetuses in the trisomy 21 group was significantly shorter than that in the control group (*p* < 0.05; Table 1).

**Table 1:** General clinical data and TL

	Control group (n = 18)	21-trisomy Group (n = 40)	t /χ^2^/z	*p*-value
Maternal age	30.28 ± 4.70	35.13 ± 5.36	-3.302	0.002^※^
Paternal age	31.50 ± 5.01	36.33 ± 5.89	-3.018	0.004^※^
Weeks of gestation	20.38 ± 2.61	19.15 ± 2.15	1.718	0.091
Occupation	16 (88.89%)	26 (65.00%)	3.546	0.054
Nationality	13 (72.22%)	31 (77.50%)	0.189	0.45
Maternal TL	1.42 ± 0.95	0.85 ± 0.54	-2.447	0.014^※^
Fetal TL	1.45 ± 0.93	0.84 ± 0.44	-2.143	0.032^※^
Paternal TL	###	0.81 ± 0.69	###	##

Note: Telomere length: TL

**Legend**: Table 1. The statistical analysis results show that there are significant differences between the two groups in maternal age and fetal TL

^※^Indicates statistically significant difference

### Maternal and fetal TL comparison between the two groups

**Fig. 1:**
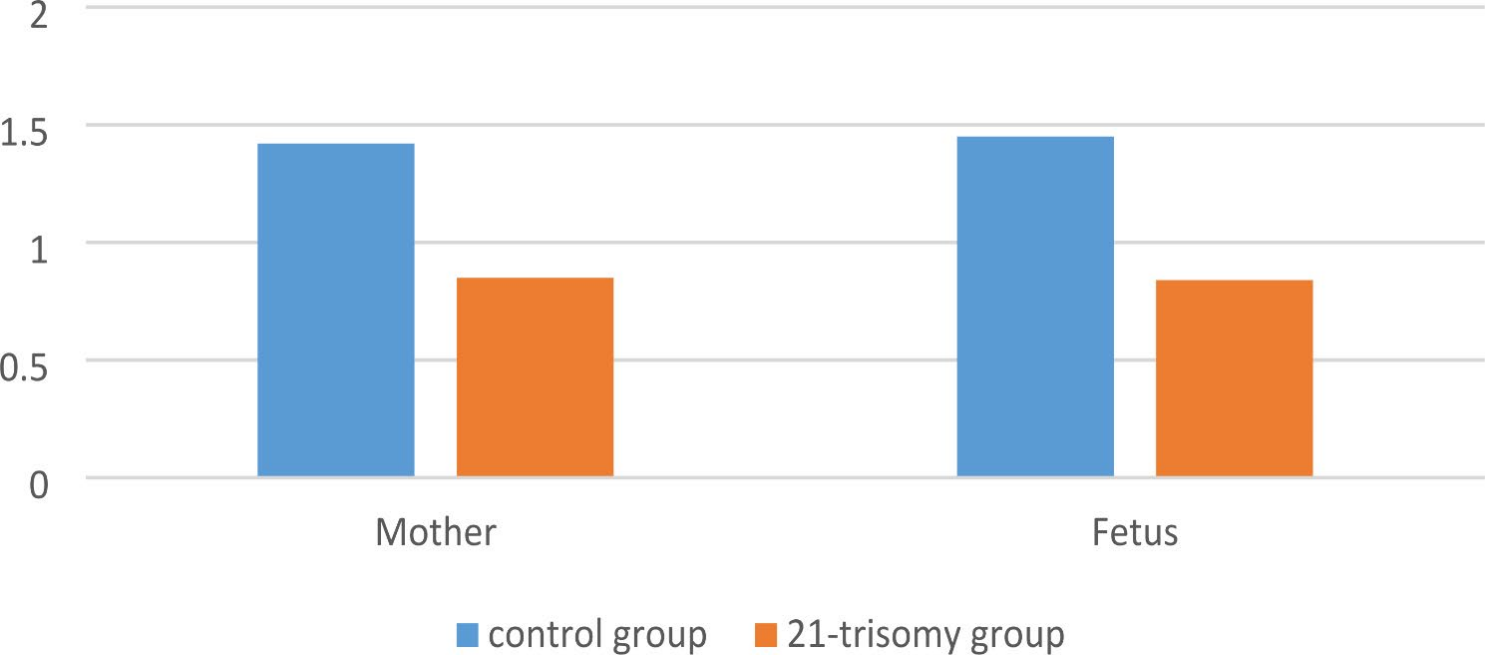
Comparison of maternal and fetal telomere length between the two groups **Legend**: Figure 1. shows that there are significant differences between the study group and the control group in the TL of mother and fetus

In the trisomy 21 group, maternal and fetal TL were significantly shorter than those in the control group (maternal, *p* = 0.014; fetal, *p* = 0.032) (Fig. 1).

**Fig. 2:**
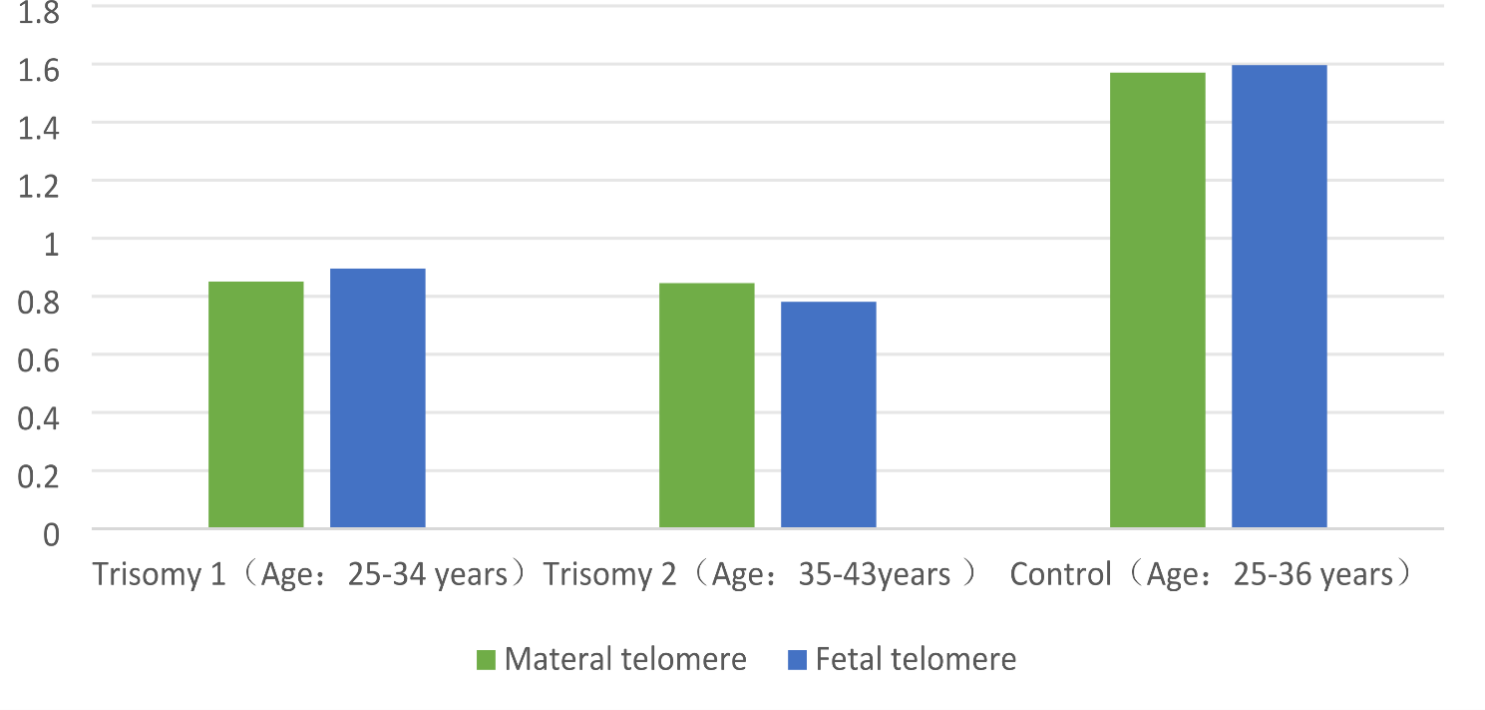
Comparison of materal and fetal telomere length between two trisomy group and control groups **Legend**: Figure 2: When compared with the control group, there were significant differences in maternal TL between the two groups. The fetal TL was significantly different only in trisomy group 2

The trisomy 21 group was divided into two groups according to the maternal age: trisomy group 1 (25–34 years) and trisomy group 2 (35–43 years). Trisomy group 1 was compared with the control group (maternal TL, 0.8505 ± 0.63467 vs. 1.42 ± 0.95, Z=-2.287, *P* = 0.022; fetal TL, 0.8963 ± 0.51671 vs. 0.84 ± 0.44, Z=-1.755, *P* = 0.079). A significant difference was observed in maternal TL between the two groups; however, no significant difference was found in fetal TL. Trisomy group 2 was compared with the control group (maternal TL, 0.8457 ± 0.45668 vs. 1.42 ± 0.95, Z=-2.362, *P* = 0.018; fetal TL, 0.7810 ± 0.35471 vs. 0.84 ± 0.44, Z=-2.361, *P* = 0.018); Significant TL differences were noted between the two groups in the mother and fetus (Fig. 2).

### Analysis of related factors affecting TL in mothers and fetuses

In the trisomy 21 group, no correlation was observed between maternal age, occupation, nationality and maternal TL. Fetal TL was positively correlated with maternal TL (r = 0.564; *p* = 0.0001) but not with maternal age, occupation, nationality, paternal age, or paternal TL (Table 2).

**Table 2:** Correlation analysis of TL between mothers and fetuses in the trisomy 21 group

MTL	Pearson correlation Sig.(two-side)	MA	occupation	nationality			
		-0.027	0.109	-0.215			
		0.868	0.502	0.182			
FTL	Pearson correlation Sig.(two-side)	MA	Occupation	nationality	MTL	PA	PTL
		-0.157	0.115	-0.02	0.564	-0.12	-0.03
		0.333	0.479	0.9	0.0001^※^	0.461	0.855

Note: MTL, Maternal Telomere Length; MA, Maternal Age; PTL, Paternal Telomere Length; PA, Paternal Age; FTL, Fetal Telomere Length

**Legend**: Table 2. The results show that there is a positive correlation between the TL of the fetus and the mother

^※^Indicates statistically significant difference

### Association between maternal TL and risk of trisomy 21

A logistic regression model was established to further investigate the association between maternal age and TL with the risk of trisomy 21 (Table 3). The results showed that maternal age and TL were associated with the risk of trisomy 21 (maternal age: OR = 1.213; 95% CI, 1.043–1.411; *P* < 0.05; maternal TL: OR = 0.311; 95% CI, 0.109–0.885, *P* < 0.05) (Table 3).

**Table 3:** Association between maternal age and TL and trisomy 21 risk in the logistic regression model

	B	S.E.	Wals	p-value	OR	OR (95% CI)
Maternal age	0.193	0.077	6.266	0.012^※^	1.213	1.043–1.411
Maternal TL	-1.169	0.534	4.795	0.029^※^	0.311	0.109–0.885
Occupation	0.77	0.447	2.972	0.085	2.16	0.9–5.183
Nationality	0.146	0.791	0.034	0.853	1.158	0.246–5.46
Constant	-5.61	2.941	3.639	0.056	0.004	

Note: TL, telomere length; OR, odds ratio; CI, confidence interval; S.E., Standard error of partial regression coefficient; B, Partial regression coefficient

**Legend**: Table 3. The results show that maternal TL shortening is a high-risk factor for trisomy 21

^※^Indicates statistically significant difference

### Accuracy of maternal age and TL in assessing the risk of trisomy 21 was analyzed using the ROC curve analysis

Comparing maternal age with maternal TL as a predictor of the trisomy 21 risk, the area under the maternal age ROC curve was 76.6%, while that of maternal TL was 66.0%. The area under the maternal age plus TL ROC curve is 80.8%. (Table 4).

**Table 4:** ROC curves for maternal age and TL to assessthe risk of trisomy 21

				95% CI	
	AUC	S.T.	P	Lower	Upper
Maternal age	0.766	0.07	0.001	0.629	0.903
Maternal TL	0.66	0.087	0.052	0.489	0.831
Maternal age + TL	0.808	0.068	0.001	0.674	0.941

Note: TL, telomere length; OR, odds ratio; AUC, area under the curve; S.E, Standard error

**Legend**: Table 4. The combined assessment of maternal age and maternal TL predicted fetal chromosome trisomy better than a single assessment

## Discussion

We observed that maternal and fetal TLs were significantly shorter in the trisomy 21 group than in the control group. Fetal TL was positively correlated with maternal TL. Notably, maternal age, occupation, and nationality did not show any significant associations with TL. It was also evident that the combination of maternal age and maternal TL provided a better prediction of fetal trisomy than the single index alone.

In this study, we compared the TLs of mothers and fetuses in the trisomy 21 with those in the control group, and the results showed that both mothers and fetuses in the trisomy 21 had shorter TLs than those the control group. This finding is consistent with that of previous studies [15, 16]. We first compared TL between the trisomy 21 and control groups; however, the difference in maternal age between the two groups may have led to the difference in TL. To further understand the effect of maternal age on TL, we further subdivided the trisomy 21 group into an advanced-age group and a young-age group and compared them with the control group. The results showed that the TL of mothers with trisomy 21 fetuses was shorter than that of the control group, both in the young and advanced-age groups. The findings suggest that maternal TL is not only affected by age but also by other factors.

We also explored factors affecting fetal TL within the trisomy 21 group, including maternal age and TL and paternal age and TL. Our findings revealed a positive correlation between fetal TL and maternal TL. However, no such correlation was observed between fetal TL and paternal age or paternal TL. In contrast, some studies have reported increased TL in the offspring of older fathers [17]. However, this study found no correlation between the father’s TL and fetal TL.

Although TL of the mother and fetus was assessed in different tissue cells in this study, there was no significant difference in TL within the same population. This finding is consistent with those of previous studies [18].

TL is determined by the combined action of genetic and environmental factors and lifestyle [19]. In the present study, we measured TL in peripheral blood lymphocytes. It can be inferred that the TL of the oocytes of women with short lymphocyte TL is also short. Short telomeres lead to chromosomal nondisjunction during oocyte meiosis, leading to trisomy 21 pregnancy [20, 21]. The maternal TL affects the fetal TL, shortening the TL in trisomy 21 fetuses.

Environmental and genetic factors and age influence an individual’s TL. Based on these results, we further investigated the effects of maternal age, occupation, and nationality on maternal TL. No correlation was found between maternal TL and these factors. Albizua et al. [9] have found that maternal TL was negatively correlated with maternal age. However, in the present study, the TL of the advanced age group was shorter than that of the younger group; the difference was not significant. Pearson’s correlation analysis showed no correlation between maternal age and TL. These varying conclusions may have resulted from a limited number of patients enrolled in this study. In addition, the TL of individuals decreases with age. Each individual is born with a different TL and lives in a different environment. Furthermore, environmental factors have an impact on telomere shortening. Environmental factors that influence TL include smoking, alcohol use, medication, exposure to toxic and harmful chemicals, and a history of chronic diseases. However, we did not analyze these factors in this study due to limited available data for statistical analysis in this small-scale study.

We established a logistic regression model to analyze the relationships between maternal age, maternal lymphocyte TL, maternal occupation, nationality, and trisomy 21. The results showed that the risk of trisomy 21 was associated with maternal age and lymphocyte telomere shortening.

We employed the ROC curve to analyze the accuracy of maternal age and lymphocyte TL as predictors of trisomy 21 risk. The area under the ROC curve for maternal age as a risk indicator was 76.5%, while that for lymphocyte TL as an evaluation index was 66.5%. It is, therefore, not ideal to evaluate the risk of trisomy 21 using the maternal lymphocyte TL index. Subsequently, when combining the maternal age with the maternal TL to predict the risk of trisomy-21, the area under the ROC curve improved to 80.8%, outperforming the single evaluation index.

Although maternal age plus telomere screening is better than single indicator screening in predicting trisomy 21 risk, it still does not achieve the desired screening effect. The reasons for the unsatisfactory performance of TL in assessing the risk of trisomy 21 may be as follows: individual variability in TLs at birth, different environmental exposures resulting in different degrees of TL wear, and potential biases in telomere detection experiments. These factors lead to inconsistent changes in TL in each individual.

Efforts should be directed towards a thorough investigation and analysis of factors affecting TL. This can improve the accuracy of telomere-based prediction of aneuploidy risk.

Currently, telomere prediction of aneuploidy risk has some limitations. However, compared with NIPT, telomere detection offers simplicity, speed, and cost-effectiveness. Telomere testing, with its relatively low cost (approximately $27 in China compared to $270 for NIPT), is suitable for first-line screening. Implementing telomere testing as a first-line screening can reduce the use of NIPT and effectively lower screening costs.

In future research, understanding more factors affecting TL change and improving telomere detection technology will contribute to the practical application of this technology in clinical settings.

## Conclusions

Mothers with trisomy 21 fetuses exhibited shorter TL than mothers with normal fetuses. However, the use of telomeres in the clinical prediction of aneuploidy risk still requires further investigation and research.

## Data Availability

The datasets used for analysis during the current study are available from the corresponding author on request.

## Abbreviations

NIPT: Non-invasive prenatal testing
TL: Telomere length
DS: Down syndrome
ROC: Receiver operating characteristic
